# Sensory Processing Profiles and Observed Behaviour: An Ecological, Function-Based Study of Challenging Behaviour in a Clinically Referred Sample of Children with Autism Spectrum Disorder and/or Intellectual Disability

**DOI:** 10.3390/bs16091561

**Published:** 2026-09-03

**Authors:** Romina Rinaldi, Axel Mayart, Marie Calista Cuykens, Eric Willaye

**Affiliations:** 1 Clinical Orthopedagogy Department, Faculty of Psychology and Educational Sciences, University of Mons, 7000 Mons, Belgium; marie.calistacuykens@umons.ac.be (M.C.C.); eric.willaye@umons.ac.be (E.W.); 2 Center for Interdisciplinary Research on Disability and Inclusion (CIRDI), University of Mons, 7000 Mons, Belgium; 3 Service d’Accompagnement Psychologique, Le CHAF—ASBL, Centre pour Personnes en Situation de HAndicap de Farciennes, 6240 Farciennes, Belgium; mayart.a@le-chaf.be

**Keywords:** autism spectrum disorder, intellectual disability, sensory processing, challenging behaviour, adaptive functioning, behavioural observation, functional assessment

## Abstract

Sensory processing differences are common in autism spectrum disorder (ASD) and intellectual disability (ID) and have been linked to maladaptive behaviour and reduced adaptive functioning, largely through caregiver-report measures. How they relate to the ecological organisation of observable challenging behaviour remains to be established. This retrospective study analysed routine care data from 70 children aged 3 to 16 years with ASD and co-occurring ID or ID alone, in which naturalistic functional behavioural assessments documented 7560 behavioural episodes. Episodes whose recorded functional hypothesis explicitly designated a sensory function were labelled sensory-motivated behaviours (SMBs), a clinically formulated hypothesis about the function of the episode. Beta-binomial models examined predictors of the proportion of episodes assigned a sensory function, and mixed-effects models accounting for the clustering of episodes within children examined the association between behavioural class and inferred function. SMBs accounted for 7.47% of episodes (565 of 7560; participant-level mean 8.19%), with substantial inter-individual variability, and because only explicit designations were retained this figure is a conservative estimate of how often practitioners attributed a sensory function. Whether the function was seeking or avoiding tracked the form of the episode, body-directed behaviours being associated with avoidance and object- or intake-directed behaviours with seeking, whereas the analyses did not detect consistent associations between the proportion of sensory episodes and either caregiver-reported sensory profiles (Sensory Profile-2) or adaptive functioning (Vineland Adaptive Behaviour Scales-II). The study thus provides an ecological benchmark for how often sensory functions are attributed in routine practice, and shows that the form of an episode carries information about its sensory direction that trait-level questionnaires do not.

## 1. Introduction

### 1.1. Sensory Processing and Behavioural Outcomes in Neurodevelopmental Conditions

Sensory processing refers to the neurological mechanisms that enable the selection, organisation, and interpretation of sensory information from the environment. It is a foundational process supporting the emergence of adaptive behaviour across typical development as maturation and experience interact (Adams et al., 2015; Cheung & Siu, 2009; Davies et al., 2009; Dunn, 1997; McCormick et al., 2016). Sensory processing differences may manifest as heightened reactivity to sensory input, reduced responsiveness to environmental cues, or an increased drive to seek sensory stimulation. These response patterns can occur across multiple sensory modalities, including visual, auditory, tactile, vestibular, oral, and multisensory domains (Miller et al., 2007).

Within autism spectrum disorder (ASD), sensory features are particularly prevalent, with estimates ranging from approximately 69% to 95% depending on the measures and populations studied, and this prevalence, together with evidence linking sensory differences to core autistic characteristics, contributed to the inclusion of sensory reactivity differences in the DSM-5 criteria for restricted and repetitive behaviours (Marco et al., 2011; Baranek et al., 2006; Baker et al., 2008; Klintwall et al., 2011; Tomchek & Dunn, 2007; American Psychiatric Association, 2013).

Sensory profile differences have been linked to lower adaptive functioning (Baker et al., 2008; Lane et al., 2010; Dellapiazza et al., 2020) and to elevated emotional and behavioural difficulties on parent-report instruments such as the Child Behaviour Checklist (Achenbach & Rescorla, 2000) and the Aberrant Behaviour Checklist (Aman & Singh, 1986), particularly irritability, hyperactivity/noncompliance, and lethargy (Nieto et al., 2017; Dellapiazza et al., 2020), including in children with autism without intellectual disability (Giacardy et al., 2018).

More granular investigations indicate that responsivity patterns are differentially associated with these outcomes: hyper-responsivity with irritability, emotional reactivity, internalising symptoms, hyperactivity, noncompliance and repetitive behaviours, and hypo-responsivity with reduced engagement with salient environmental cues and diminished social reciprocity (Ben-Sasson et al., 2009; Hilton et al., 2010; Boyd et al., 2010). These associations are partly explained by caregiver-reported regulatory and cognitive difficulties (Fernandez-Prieto et al., 2021), and early profiles account for variance in later outcomes (Fabbri-Destro et al., 2022). At the quadrant level of the Child Sensory Profile-2 (Dunn, 2014), sensory avoiding is most strongly associated with behavioural difficulties on the Aberrant Behaviour Checklist and uniquely predicts caregiver strain (Griffin et al., 2022).

Beyond autism, sensory responsivity differences are consistently documented across genetic neurodevelopmental conditions, including Williams, Down, and fragile X syndromes, and in intellectual disability not attributable to a specific syndrome. Across these populations they are highly prevalent and associated with lower adaptive functioning, behavioural difficulties, and reduced everyday participation, whether indexed through standardised caregiver-report questionnaires (John & Mervis, 2010; Will et al., 2019; Lachiewicz et al., 2024; Engel-Yeger et al., 2011) or, more recently, through direct psychophysical assessment of tactile reactivity (Gunderson et al., 2025).

Collectively, this literature indicates that sensory processing differences are consistently associated with adaptive functioning, emotional symptoms, and behavioural difficulties across neurodevelopmental populations. Adaptive functioning is of particular interest in this context, not only as a correlate of sensory processing but as a plausible predictor of how sensory-related needs are behaviourally expressed. Children with more limited communication, daily-living, and self-regulatory skills have fewer conventional means to obtain, avoid, or modulate sensory input, and may therefore rely more heavily on challenging behaviour to do so, whereas stronger adaptive skills may afford alternative, less disruptive strategies (Carr & Durand, 1985). Patterns of sensory responsiveness explain unique variance in distinct VABS-II domains, sensory seeking being associated specifically with communication and hyperresponsiveness with daily living skills and socialisation (Feldman et al., 2020), while sensory avoiding has been linked to daily living skills in preschool children with autism (Jasmin et al., 2009). Three-year change in sensory profile has likewise been accompanied by change in socialisation and daily living skills rather than in communication (Dellapiazza et al., 2021). Communication is thus expected to bear most directly on the availability of alternatives for requesting or refusing sensory input, and daily living skills on the capacity to organise and tolerate the sensory demands of everyday routines.

### 1.2. Limits of Questionnaire-Based Evidence

Despite this growing body of evidence, most of this literature relies predominantly on caregiver-report questionnaires (e.g., the Short Sensory Profile, the Aberrant Behaviour Checklist, and the Child Behaviour Checklist), which capture the frequency and severity of behavioural features but offer only indirect insight into the contextual conditions under which behaviours occur; associations between sensory responsivity and behaviour are therefore typically inferred rather than directly observed. This limitation is especially salient for challenging behaviours that pose significant risk, such as self-injury, aggression, and property destruction, and that are frequent, severe, and embedded within complex clinical contexts.

### 1.3. Sensory-Motivated Behaviours: Definition and Delimitation

Against this backdrop, this study focuses on sensory-motivated behaviours (SMBs). An SMB is defined at the level of the observed episode and by the sensory nature of the event held to control it, a relation inferred by the practitioner rather than demonstrated experimentally, and not by the topography of the behaviour. Two patterns fall under this definition: (i) that the behaviour itself produced an immediate and identifiable change in stimulation, which constitutes its own product and requires no other person to deliver it, or (ii) that it was occasioned by an aversive sensory event present in the setting (e.g., noise, bright light, crowding, unexpected physical contact) and was followed by the termination of that event or by the child’s removal from it. The first pattern corresponds to sensory seeking, the second to sensory avoiding.

The idea that a behaviour may function to produce sensory consequences is not new. Within applied behaviour analysis, behaviours maintained by the sensory stimulation they generate, independently of social consequences, are described as automatically reinforced, or sensory-maintained (Iwata et al., 1994; Hanley et al., 2003). Sensory-motivated behaviours are introduced here as an operational category derived from this established functional class, from which they differ in what can be claimed about them: the subset of challenging behaviours whose inferred function, in the present data, is to modify the individual’s sensory input. The term motivated, rather than maintained, is used deliberately. Maintenance names a demonstrated reinforcement relation, whereas an antecedent–behaviour–consequence record documents what preceded and followed an episode. A sensory-motivated behaviour is therefore a clinical hypothesis about the function of an episode, formulated by the practitioner who observed it. This functional framing also distinguishes sensory-motivated behaviours from neighbouring constructs that are primarily descriptive. Sensory-seeking and sensory-avoiding profiles characterise the direction of an individual’s sensory responsivity rather than the function of any particular behaviour, whereas restricted and repetitive behaviours are defined by their form and only partly overlap with behaviours that serve a sensory function (Boyd et al., 2010). Sensory-motivated behaviours thus cut across these topographies, grouping behaviours that differ in appearance but share an inferred sensory function.

Sensory motivation and automatic reinforcement pick out overlapping but distinct sets of behaviours. Automatic reinforcement is defined by the absence of social mediation (Vaughan & Michael, 1982; Vollmer, 1994), and sensory seeking generally falls within it, since the stimulation produced is inseparable from the behaviour. Sensory avoiding sits astride the boundary: the aversive stimulation is often removed by a caregiver responding to the episode, so the contingency is socially mediated even where the controlling event is sensory. Both patterns belong to a single category here because the classification follows the sensory nature of that event rather than the mechanism by which the change in stimulation is obtained. On the same principle, sensory avoiding is distinguished from escape, attention and access to tangibles by what is terminated or delivered: a sensory event present in the setting, rather than a demand, a task, a transition, or an outcome supplied by another person independently of the child’s sensory environment.

Pain, anxiety, and emotional regulation belong to a different logical level: they are motivating operations or setting events that alter the value of a consequence and the momentary likelihood of behaviour (Michael, 1993; Kennedy & Meyer, 1996; Carr & Owen-DeSchryver, 2007) rather than classes of reinforcement, may accompany behaviour serving any of the functions above, and are therefore treated as contextual conditions rather than as competing functional categories. Finally, a sensory attribution was never used as a residual category for episodes in which no other explanation was apparent, a default use long criticised (Kennedy, 1994).

### 1.4. Attributing Function from Descriptive Records

Because sensory-motivated behaviours rest on functional hypotheses formulated in the course of care, the accuracy of such hypotheses bears on what the category can support, and it has been studied empirically. Correspondence between descriptive and experimental functional analyses is generally poor: Lerman and Iwata (1993) obtained agreement for 1 of 6 individuals with self-injury, and Thompson and Iwata (2007) found the most frequently observed consequence to be the maintaining variable in only 3 of 12 cases, attention being the recurrent source of error, confirmed as maintaining for only 2 of the 8 participants for whom it was the most frequently observed consequence (see also Mace et al., 1991; St. Peter et al., 2005). Correspondence improves when both forms of assessment are conducted in the setting where the behaviour occurs, whether by teachers running descriptive and classroom experimental analyses (Sasso et al., 1992) or through trial-based functional analysis, which matched a standard functional analysis in 6 of 10 cases (Bloom et al., 2011; see also Rispoli et al., 2014). Functional analysis outcomes themselves vary with the setting in which they are obtained (Lang et al., 2008, 2010), and contrived settings may omit the evocative or discriminative stimuli that occasion behaviour in everyday environments. The two procedures therefore sample different conditions, and establishing a functional relation for an individual requires experimental analysis.

The analyses reported below therefore concern the distribution of sensory attributions across episodes and children rather than the function of any individual episode.

### 1.5. Aims and Research Questions

Using observational data collected in intensive intervention contexts with children with ASD and intellectual disability, and with children with intellectual disability without autism, the study does three things: it quantifies how often a sensory function is attributed to challenging behaviour in routine practice, it tests whether caregiver-reported sensory profiles predict that attribution, and it examines how sensory functions are organised across behavioural forms.

The study is guided by the following overarching research question:

General Research Question (RQ0): To what extent are established associations between sensory processing profiles and challenging behaviours observable in a clinically complex sample of children with severe behavioural difficulties, and how can these associations be characterised through direct ecological observation?

This overarching aim is operationalised through three sub-questions. The third is treated as the primary analysis, the remaining questions and the class-by-function models as exploratory.

RQ1: Can sensory-motivated behaviours be identified through ecological observation in this clinical sample, and how are they distributed in terms of frequency, behavioural form, and inferred sensory function? Does their relative occurrence vary descriptively with age or gender? These comparisons are exploratory.

RQ2: Within this sample, do caregiver-reported sensory processing profiles (Sensory Profile-2) relate to caregiver-reported behavioural difficulties (Nisonger Child Behaviour Rating Form), as prior questionnaire-based research would predict?

RQ3: Do caregiver-reported sensory responsivity profiles (Sensory Profile-2 quadrants) and adaptive functioning predict the proportion of episodes assigned each sensory function through direct observation, and do these predictors operate differently for sensory-seeking and sensory-avoiding behaviours?

On the basis of these constructs, we anticipated theoretically corresponding relationships between sensory responsivity profiles and SMB subtypes: the Sensation Seeking quadrant was expected to relate most strongly to sensory-seeking SMBs, and the Sensation Avoiding and Sensory Sensitivity quadrants to sensory-avoiding SMBs, whereas Low Registration, reflecting elevated neurological thresholds, was expected to relate to sensory-seeking behaviours undertaken to augment sensory input. Higher adaptive functioning was expected to be associated with fewer SMBs, reflecting a greater availability of alternative regulatory and communicative strategies. We further expected sensory seeking and sensory avoiding to be unevenly distributed across behavioural forms, since the sensory consequence a behaviour can produce depends on what the behaviour does: behaviours that act upon the body or upon another person afford the termination of stimulation, whereas behaviours that act upon objects or upon oral intake afford its production.

## 2. Materials and Methods

### 2.1. Research Design and Data Collection

This study employed a retrospective design based on Routine Care Data (RCD) extracted from an intensive behavioural intervention service operating across four multidisciplinary mobile teams of the SUSA Foundation (Service Universitaire Spécialisé pour personnes avec Autisme), Belgium. The service provides specialised support for children, adolescents, and young adults who present with severe challenging behaviours and have a diagnosis of ASD and/or intellectual disability. Each team comprises highly trained professionals, including psychologists, physicians, speech and language therapists, and support workers, all with specialist expertise in challenging behaviour and functional assessment. Interventions are delivered directly within individuals’ daily environments (e.g., home, school, residential or day-care services), and standardised procedures and reporting templates are used consistently across teams.

RCD were extracted from the service’s secure clinical database for all cases supported between 1 January 2018 and 31 March 2025. The intervention model follows a structured, multi-cycle process comprising four phases: (1) a systematic observation phase conducted across the individual’s environments over a three-month period; (2) an intervention phase informed by functional hypotheses; (3) training and sensitisation of caregivers and professionals; and (4) continued or tapered intervention when clinically indicated. Only data from Phase 1 (pre-intervention) were included in this study’s analyses.

Eligibility for intervention by the service required a documented diagnosis of ASD and/or intellectual disability, established outside the service, together with recurrent, observable behaviours posing a significant risk to the individual or others, either through physical harm (e.g., self-injury, aggression, ingestion of non-food substances) or through substantial psychological impact on caregivers or peers. Behaviours also had to cause persistent and marked disruption to daily activities or social participation, including jeopardising the individual’s ability to remain in their home, educational placement, or care setting despite external support, to occur at least weekly (unless actively prevented), to persist for a minimum of three months, and not to be attributable to substance use or an acquired neurological condition.

During Phase 1, all eligible clients completed descriptive functional behavioural assessments (O’Neill et al., 2015; Cooper et al., 2020), that is, systematic identification of the contextual variables that evoke and maintain challenging behaviour. In this service, assessments relied on Antecedent–Behaviour–Consequence (ABC) descriptive procedures implemented directly within the client’s natural environments, with real-time recording of antecedent events, behavioural topographies, and immediate consequences, thereby providing ecologically valid information about naturally occurring contingencies (Hanley et al., 2003).

Observation exposure was determined by the structure of the intervention cycle rather than by individual scheduling. As described above, Phase 1 comprises a three-month observation period conducted across the individual’s environments, and this structure is identical for every client; only children who completed the phase were retained (see Section 2.3). The period during which behavioural episodes could be documented was therefore equivalent across participants by design. Within that period, however, observation intensity cannot be quantified: the service did not record the number of observation sessions, the duration of individual episodes was not captured, and no measure of recording coverage was maintained. The assessment files include fields for the date and the setting of each episode, completed at the discretion of the practitioner. The date field was left blank at entry for most children, so episodes cannot be grouped into sessions, whereas the setting field is populated for almost all episodes and records several distinct settings per child. Differences in staffing, living arrangements, and opportunities for observation may consequently have influenced how many episodes were documented for a given child, independently of how often challenging behaviour actually occurred.

Practitioners were trained within the programme and worked from a common assessment framework and terminology, and a limited set of formulations recurs across teams and years.

### 2.2. Data Preparation

A data preparation framework was developed to support the systematic processing of behavioural data derived from the descriptive functional assessments. Framework development was undertaken collaboratively with professionals from the intervention unit to ensure alignment with routine clinical practices and terminology.

The first stage of data preparation involved harmonising the lexical forms used to describe behaviours. As functional assessments were completed by multiple practitioners, the same behaviour could be labelled using different terms (e.g., shouting, screaming, howling). In consultation with the clinical teams, these variations were consolidated under a single behavioural identifier corresponding to a unique item number (e.g., all forms of shouting were mapped to item 810).

In the second stage, behaviours were organised into broader behavioural categories, yielding eight categories to which each record was assigned once and only once: Self-Injurious Behaviours (SIB, e.g., self-biting, head-hitting); Aggression towards Others (AO, e.g., hitting, biting, scratching); Socially in Appropriate Behaviours (SAB, e.g., absconding or escaping, dropping to the floor, inappropriate touching, spitting); Eating-related Behaviours (ED, e.g., pica involving oral exploration or ingestion, persistent food seeking); Sexualised Behaviours (SEXUALISED, e.g., public masturbation, inappropriate touching of others); Destruction of Materials (DM, e.g., breaking or knocking down objects); Stereotyped Behaviours (STEREOTYPED); and Behavioural Crises or Meltdowns (MELTDOWNS), operationalised as episodes comprising multiple behaviours from different categories occurring simultaneously over a circumscribed period and attributable to a single inferred function.

Because this last category operates at a different level of aggregation from the others, the unit of analysis requires specification. Categories were applied to records as entered by practitioners at the time of observation, and episodes were not re-segmented by the research team, so the unit is the record line, that is, a single entry in the functional assessment file, several of which may arise from a single situation. The exception is the crisis category, used routinely in the service for the co-occurrence of several behavioural topographies within a very short time window attributable to a single inferred function, documented on one line. Mutual exclusivity accordingly holds at the level of the record but not at the level of behavioural topography, so counts for the topographical categories exclude behaviours occurring within crises and under-represent the total occurrence of those topographies, and the function attached to a crisis applies to the episode as a whole rather than to its constituent behaviours. Four of the eight categories correspond to topographies routinely distinguished in the challenging-behaviour literature, namely self-injury, aggression, destruction of materials and stereotyped behaviour (Emerson, 2001); the remaining four, socially inappropriate, eating-related and sexualised behaviours and behavioural crises, are categories used routinely in the service. What varied across teams and years was the wording used to describe individual behaviours, and these descriptors were consolidated, jointly with the clinical teams, into the eight categories. Category definitions and a worked example of the crisis unit are given in Table S8.

A parallel data preparation procedure was applied to inferred behavioural functions. Sensory-related functions were grouped into two categories: seeking sensory stimulation or avoiding sensory stimulation. This dichotomy reflected the fact that sensory functions in the dataset were consistently described using these two forms and aligns with the obtain/avoid framework proposed by Matson et al. (2011).

Functional hypotheses were recorded by the practitioner who conducted the observation, as an integral part of the functional assessment record, and were not re-inferred by the research team. The assessment template used by the service specifies a set of functions from which practitioners work, so the hypotheses are not free-form interpretations, although the wording entered was not standardised. The research coding takes the recorded hypothesis as given without revisiting the contingency. It proceeded in two steps: semantic variants designating the same function were consolidated under a single code (e.g., “sensory stimulation seeking” and “sensory seeking”), and a behaviour was coded as sensory-motivated only when the recorded hypothesis explicitly designated sensory seeking or sensory avoidance, the hypothesis being coded as non-sensory in the absence of an explicit reference to the sensory domain. This restrictive rule avoids attributing functions that had not been stated by the practitioner who observed the episode. Hypotheses designating self-stimulation, which do not in themselves specify a sensory function, required an additional rule: they were coded as sensory seeking only when the antecedent recorded for the episode referred to sensory stimulation, and as non-sensory otherwise. Coding was carried out manually, line by line, by one author (A.M.), without automated text matching, and the hypothesis as originally recorded was retained alongside the assigned code. The full decision rules, the distinction between the practitioners’ and the research level of decision, and the arguments for and against the self-stimulation rule are set out in Table S8.

Reliability of the research coding. The first coder (A.M.) is an author of this manuscript and was not blind to the original coding decisions, so the research coding step was recoded independently by a second coder (M.C.C.). A stratified random sample of 500 records was drawn (6.6% of the corpus; seed 20260810), comprising 250 records originally coded as non-sensory, 106 as sensory-seeking and 144 as sensory-avoiding, and covering 63 of the 70 children; the sample was enriched in sensory codes so that agreement could be estimated on the two rare categories. Records were presented without the original codes, together with the coding rules reproduced in Table S8, and each was assigned one of the three function codes. Agreement is reported as Cohen’s κ (Cohen, 1960) with a percentile bootstrap confidence interval (2000 resamples), and, because κ is depressed when one category dominates, a pattern documented as the kappa paradoxes (Feinstein & Cicchetti, 1990), as Gwet’s AC1 (Gwet, 2008) and the prevalence-adjusted bias-adjusted kappa (Byrt et al., 1993), which are less sensitive to the marked imbalance of the function variable, and per category. Estimates refer to the recoded sample rather than to the base rates of the corpus. The recoding covers one step, the research coding of hypotheses already recorded. The hypotheses themselves, the delimitation of episodes and their assignment to behavioural categories were produced in routine care and could not be reassessed.

Two consequences follow. The classification is inherited from clinical judgements made in situ, so its validity depends on the practitioners’ functional hypotheses. And because only explicit sensory designations were retained, the resulting count of sensory-motivated behaviours is conservative: episodes whose function may have been sensory but was recorded in non-sensory or unspecified terms were classified as non-sensory. The proportion of sensory-motivated behaviours reported below should therefore be read as a conservative estimate of the sensory functions practitioners explicitly recorded.

### 2.3. Sample

Inclusion criteria for this study were as follows: (a) children and adolescents aged 16 years or younger at the time of assessment, the observed range being 3 years 4 months to 16 years 11 months; (b) a clinical diagnosis of ASD with co-occurring intellectual disability, or intellectual disability alone; (c) absence of perceptual disorders such as visual or auditory impairments; and (d) availability of complete behavioural data and of a caregiver-reported sensory assessment (see instruments). The Nisonger Child Behaviour Rating Form, French version (NCBRF; Tassé et al., 1999), is validated from 3 to 16 years. The Child Sensory Profile-2 is validated from 3 years 0 months to 14 years 11 months, and four participants exceeded that upper limit; all models were repeated without them as a sensitivity check (Table S5). Children with ASD and co-occurring intellectual disability and children with intellectual disability alone were analysed together, since sensory responsivity differences are documented in both populations and sensory processing patterns have been shown to predict behavioural outcomes over and above the variance attributable to diagnostic grouping (Schulz et al., 2023).

Diagnoses were not established by the intervention service. Admission to the programme is conditional on a documented diagnosis of ASD, of intellectual disability, or of both, issued or validated by a psychiatrist or a specialist diagnostic centre, so that all diagnoses predate the assessments analysed here. The instruments and criteria applied were those of the referring services and are not recorded in the programme’s files.

Severity of intellectual disability was recorded whenever it was documented by a physician in the medical section of the clinical database or by the regional disability classification, and coded as unspecified otherwise, which was the case for 47.15% of the sample. No standardised cognitive assessment was administered within the programme, and no IQ or developmental quotient data were available in the records.

A total of 313 clients supported during the target period were initially identified. Clients who had not completed the observation phase of the intervention cycle were excluded first (*n* = 15; 4.79%). An interrupted phase yields a shorter and non-comparable recording window (Section 2.1). Interruptions arose from changes in service indication, reorientation to another service, or discontinuation of follow-up, and were unrelated to the behaviours under study. Individuals who did not meet the age criteria were then excluded (*n* = 178; 56.87%). Finally, clients without available sensory processing assessments were excluded (*n* = 50; 15.97%), as sensory data were required to address the study’s research questions. The final sample therefore comprised 70 individuals (see Table 1) with complete functional and sensory datasets. Across this sample, 7560 behavioural episodes and their associated functional descriptors were processed.

Administration of the Sensory Profile-2 was not systematic within the programme: the instrument was completed when the assessing clinician judged a sensory evaluation to be indicated in view of the child’s profile and the presenting concerns. Completed protocols were also archived by the psychologist who had administered them rather than encoded centrally, and some could no longer be retrieved after professional turnover over the seven-year period.

### 2.4. Instruments

As part of the service’s standardised multidisciplinary assessment protocol, several caregiver-reported standardised measures are routinely collected. These instruments were not administered specifically for research purposes; rather, all available parent-reported questionnaires were retrieved from the clinical database. Because these data were drawn from routine clinical records, only scale-level summary scores were available rather than item-level responses; internal-consistency reliability could not be estimated within the present sample. These are, however, standardised and validated instruments whose reliability is documented in their respective manuals (Dunn, 2014; Tassé et al., 1999; Sparrow et al., 2005).

#### 2.4.1. Dunn’s Sensory Profile Second Edition (Sensory Profile-2), French Version

Sensory processing was assessed using the French version of Dunn’s Sensory Profile-2 (Dunn, 2014), completed by caregivers. The instrument is based on Dunn’s (1997, 2007) model of sensory processing, in which sensory behaviours emerge from the interaction between an individual’s neurological threshold, low corresponding to hyperreactivity and high to hyporeactivity, and the strategies used to regulate sensory input, either active or passive. The intersection of these constructs yields four sensory processing patterns: Sensation Seeking, Low Registration, Sensory Sensitivity, and Sensation Avoiding.

#### 2.4.2. Nisonger Child Behaviour Rating Form (NCBRF), French Version

Challenging behaviours were documented using the Nisonger Child Behaviour Rating Form, French version (NCBRF; Tassé et al., 1999), a standardised caregiver-report measure designed for children and adolescents with intellectual disability and related neurodevelopmental conditions. The NCBRF comprises eight scales, namely: Conduct Problem, Insecure/Anxious, Hyperactive, Self-Injury/Stereotypic, Self-Isolated/Ritualistic, Overly Sensitive, Compliance/Calm, and Adaptive Social. In this study, the NCBRF served two descriptive purposes: to characterise the sample’s behavioural profile (Table 1) and to examine the convergence between caregiver-reported sensory processing and caregiver-reported behavioural difficulties (RQ2). Descriptive statistics are reported for all eight scales, whereas the correlational analyses focused on the six maladaptive behaviour subscales.

#### 2.4.3. Vineland Adaptive Behaviour Scales, Second Edition (VABS-II), French Version

Adaptive functioning was documented using the Vineland Adaptive Behaviour Scales, Second Edition (VABS-II; Sparrow et al., 2005), a widely used instrument that measures communication, socialisation, daily living skills, and motor abilities through semi-structured interviews with caregivers. The French VABS-II was used in preference to the latest version (VABS-III), as it was specifically adapted and validated for French-speaking populations while retaining the structure of the original instrument.

Three scoring metrics were derived: raw subdomain scores summed within each of the three domains (Communication, Daily Living Skills, and Socialisation) and across domains; v-scale scores, which are age-referenced, obtained from the exact age at assessment; and the domain standard scores and Adaptive Behaviour Composite derived from them. The norm-referenced metrics behave very differently in this sample. The composite was at the floor value of 20 for 44 of the 50 participants for whom it could be computed, and the Communication and Socialisation standard scores for 44 and 45 of 52, respectively, so the composite cannot serve as a continuous measure here. The sums of v-scale scores retain their variance (range 9 to 75), because the compression occurs at the conversion step, in which a v-scale sum anywhere between 3 and 14 maps onto a single standard score of 20. The sum of v-scale scores was therefore retained for the primary analysis, since it is age-referenced and preserves the variance that the standard scores lose, and the summed raw score, which is not adjusted for age, is reported alongside it as a sensitivity analysis.

Summing the three domains was examined rather than assumed, and is supported by the internal consistency of the v-scale sums (α = 0.756, first principal component 68% of the variance) and by the instability of the coefficients when the three domains are entered simultaneously. The derivation of each metric, the floor distributions per metric, and these summation diagnostics are reported in Table S6.

### 2.5. Statistical Analysis

All statistical analyses were conducted using JASP (version 0.18.3.0; JASP Team, 2024) and R (version 4.3.3; R Core Team, 2024). Descriptive statistics were first computed to characterise the distribution of observed challenging behaviours across predefined behavioural and functional categories.

Missing data affected two instruments. On the NCBRF, one participant’s eight subscale values were non-integer and therefore imputed at an earlier stage, without documentation of the procedure; that participant was excluded from all NCBRF analyses, which are based on 69 participants. All remaining variables other than the VABS-II were complete, missingness on that instrument reflecting the fact that some clients did not undergo the assessment as part of routine clinical practice. Multiple imputation by chained equations was performed for the 18 missing values (25.7%) (Rubin, 1987; van Buuren & Groothuis-Oudshoorn, 2011), following current guidance on the number of imputed datasets and the specification of the imputation model (White et al., 2011); the imputation model, the number of datasets and iterations, and the convergence checks are reported in Table S4.

To quantify the individual contribution of sensory factors, a variable was derived representing the proportion of sensory-motivated behaviours per participant. Given its bounded, non-normal distribution, non-parametric tests were used in all group comparisons and correlational analyses conducted in JASP: a Spearman rank correlation for age and a Mann–Whitney U test for gender. Diagnostic groups were not compared, the intellectual disability-only subgroup comprising 10 participants and the study being designed to characterise a clinically referred population rather than to test diagnostic contrasts.

To examine whether sensory processing profiles and adaptive functioning predicted the proportion of episodes assigned each sensory function, beta-binomial regression models (Crowder, 1978) were fitted separately for sensory-seeking and sensory-avoiding episodes. The estimand is the proportion of a child’s documented episodes that were assigned a sensory function, with the number of sensory episodes as the numerator and the child’s total number of documented records as the denominator; the observation window was fixed and identical for all participants, though observation intensity within it was not recorded, so the outcome is the proportion of a child’s documented records assigned a sensory function. The beta-binomial specification was required by the data rather than chosen for convenience: both outcomes were substantially overdispersed under a binomial model, which biases standard errors when left unmodelled (Bolker et al., 2009; Harrison, 2015); the beta-binomial improved fit in each case, and it also absorbs part of the clustering of episodes within children, which a binomial model would ignore (Table S3). Three predictors were entered per model, prespecified from the directional hypotheses stated in the Introduction: Sensation Seeking, Low Registration, and adaptive functioning for sensory seeking; Sensation Avoiding, Sensory Sensitivity, and adaptive functioning for sensory avoiding. The restriction to three predictors follows from the number of available events rather than from collinearity, which the diagnostics show not to be limiting. Because episodes are clustered within children, which violates the independence assumption of fixed-effects models (Bolker et al., 2009), the association between behavioural class and inferred function was analysed at the episode level using mixed-effects logistic models with a random intercept for participant. Two models were fitted, whether an episode was assigned a sensory function and, among sensory episodes, whether that function was avoidance rather than seeking, with intraclass correlations reported for both, computed on the latent-variable scale (Nakagawa et al., 2017). Socially inappropriate behaviours served as the reference class in both models, being the most frequent class and therefore the most precisely estimated baseline; this choice affects the parameterisation of the contrasts but not model fit. A model containing all four quadrant scores together with adaptive functioning is reported as an exploratory analysis (Table S2). Model diagnostics and sensitivity analyses, which added age and then gender and repeated the models after excluding participants beyond the validation range of the Child Sensory Profile-2, are reported in Tables S3 and S5, respectively. Model fitting used mice 3.16.0 (van Buuren & Groothuis-Oudshoorn, 2011) and glmmTMB 1.1.8 (Brooks et al., 2017), with a fixed random seed.

One analysis was designated as primary: the association between caregiver-reported sensory responsivity, adaptive functioning, and the proportion of episodes assigned a sensory function. All other analyses, including the comparisons by age and by gender, the class-by-function models, and the correlations between caregiver-reported instruments, are exploratory and are reported as such. Where multiplicity is at issue, uncorrected *p*-values are reported alongside corrected ones: Bonferroni within each family of class contrasts, and Holm (1979) and Benjamini and Hochberg (1995) for the 24 correlations. Effect sizes are reported with confidence intervals throughout; intervals for rank-based statistics were obtained by percentile bootstrap with 5000 resamples (Efron & Tibshirani, 1993). A nominal alpha of 0.05 is used as a reference point rather than as a decision rule, and no conclusion rests on a *p*-value crossing that threshold.

## 3. Results

### 3.1. Distribution of Challenging Behaviours

Results are reported in relation to the study’s research questions. We first describe the observed challenging behaviours and the identification and distribution of sensory-motivated behaviours (RQ1), then examine the convergence between caregiver-reported sensory profiles and behaviour (RQ2), and finally model the proportion of episodes assigned each sensory function (RQ3). A total of 7560 challenging behaviours were identified in the dataset and processed across the sample (Table 2). Behaviours were categorised into eight mutually exclusive behavioural classes (see data coding section). Socially inappropriate behaviours were the most frequent category (*n* = 3340; 44.18%), encompassing behaviours such as spitting, absconding, and dropping to the floor. This was followed by aggression towards others (*n* = 1685; 22.29%) and destruction of materials (*n* = 1146; 15.16%). Self-injurious behaviours, meltdowns, eating-related behaviours, sexualised behaviours, and stereotyped behaviours occurred less frequently.

On the 500 recoded records, the two coders agreed on 459 (91.8%), κ = 0.86, 95% CI 0.82 to 0.90. Gwet’s AC1 was 0.88 and the prevalence-adjusted bias-adjusted kappa 0.88. Per-category agreement was 0.94 for sensory avoiding and 0.79 for sensory seeking. Of the 41 disagreements, 28 concerned hypotheses designating self-stimulation for which the recorded antecedent was ambiguous as to its sensory character, and 9 concerned hypotheses designating the avoidance of an unspecified constraint for which the antecedent, but not the hypothesis itself, referred to a sensory event.

### 3.2. Distribution of Sensory-Motivated Behaviours (RQ1)

#### 3.2.1. Frequency of Sensory Attributions

Two quantities are reported throughout, since they weight children differently. Pooled across all recorded episodes, 565 of 7560 episodes (7.47%) were labelled as sensory-motivated, of which 338 (59.82%) as sensory avoidance and 227 (40.18%) as sensory seeking; this event-level percentage is dominated by the children who contributed the largest numbers of episodes. Averaged across children instead, the mean participant-level proportion was 0.082 (*SD* = 0.113), that is 8.19% (*SD* 11.35) of a given child’s episodes, with a median of 0.037 (IQR 0 to 0.108). This distribution was strongly right-skewed (Shapiro–Wilk W = 0.715, *p* < 0.001): 20 of the 70 children (29%) had no episode classified as sensory-motivated, whereas the highest observed proportion was 0.481. Sensory-motivated episodes were recorded in every behavioural class, and the proportion of episodes assigned a sensory function ranged from 25.5% of sexualised behaviours to 1.5% of behavioural crises (Table 2).

#### 3.2.2. Behavioural Class and the Assignment of a Sensory Function

Whether an episode was assigned a sensory function was associated with behavioural class, χ^2^(7) = 45.93, *p* = 9.07 × 10^−8^, with 44.1% of the variance in that outcome located between participants (ICC = 0.441). With socially inappropriate behaviours as the reference class, sexualised behaviours were more likely to be assigned a sensory function (OR = 4.04, 95% CI 2.26 to 7.20, *p* < 0.0001), whereas self-injurious behaviours (OR = 0.67, 95% CI 0.47 to 0.96, *p* = 0.030) and behavioural crises (OR = 0.24, 95% CI 0.07 to 0.77, *p* = 0.017) were less likely. Across the seven comparisons, only the contrast for sexualised behaviours survives Bonferroni correction (α = 0.007); the remaining two are reported as exploratory. Full model output is reported in Table S9, panel A.

#### 3.2.3. Behavioural Class and Sensory Seeking Versus Sensory Avoiding

Within the 565 episodes assigned a sensory function, whether that function was seeking or avoiding, hereafter its direction, also varied by behavioural class (χ^2^(6) = 125.63, *p* < 2.2 × 10^−16^; ICC = 0.762). Behavioural crises and stereotyped behaviours, each contributing fewer than ten sensory episodes, were combined into an “other” category. Relative to socially inappropriate behaviours, sensory avoiding rather than sensory seeking predominated for aggression towards others (OR = 7.42, 95% CI 2.62 to 20.99, *p* = 0.0002) and for self-injurious behaviours (OR = 5.32, 95% CI 1.87 to 15.13, *p* = 0.0017), whereas sensory seeking predominated for destruction of materials (OR = 0.12, 95% CI 0.04 to 0.34, *p* < 0.0001), sexualised behaviours (OR = 0.03, 95% CI 0.003 to 0.28, *p* = 0.0024) and eating-related behaviours (*p* = 0.0032; the point estimate is not reported, as it rests on a single episode in one cell). Five of the six comparisons survive Bonferroni correction (α = 0.0083); full model output is reported in Table S9, panel B.

### 3.3. Exploratory Associations of Sensory-Motivated Behaviours with Age and Gender (RQ1)

The Spearman correlation revealed a non-significant negative association between age and the proportion of SMBs (ρ = −0.211, *p* = 0.079). The estimate was negative and imprecise, and in a beta-binomial model age was not associated with the proportion of sensory episodes (OR per year of age 0.937, 95% CI 0.852 to 1.032, *p* = 0.187). The proportion of episodes assigned a sensory function was higher in girls (median 0.108, IQR 0.068 to 0.122) than in boys (median 0.026, IQR 0 to 0.093), and the difference was specific to sensory avoiding (girls: median 0.086, IQR 0.050 to 0.118, none at zero; boys: median 0.006, IQR 0 to 0.057, 48% at zero; rank-biserial −0.616, 95% CI −0.794 to −0.407, *p* = 0.0001), being absent for sensory seeking (rank-biserial −0.081, 95% CI −0.356 to 0.193, *p* = 0.573). In a beta-binomial model including gender and age (*n* = 70), the association with sensory avoiding held (OR = 3.17, 95% CI 1.84 to 5.47, *p* < 0.0001), with no association for sensory seeking. The difference reflects presence rather than frequency: none of the 16 girls had zero episodes of sensory avoiding, against 26 of the 54 boys (Fisher’s exact *p* = 0.00023). This comparison was not planned and rests on 16 girls; it is reported as exploratory.

### 3.4. Associations Among Caregiver-Reported Measures (RQ2)

Correlation analyses were conducted to examine associations between caregiver-reported sensory processing patterns and behavioural difficulties. Across the 24 Spearman correlations between the four Sensory Profile-2 quadrants and the six maladaptive subscales of the Nisonger Child Behaviour Rating Form (*n* = 69), only one reached nominal significance, between Sensation Avoiding and the Self-Injury/Stereotypic subscale (ρ = 0.240, 95% CI −0.007 to 0.457, *p* = 0.047); this association survived neither Holm nor Benjamini–Hochberg correction. The median absolute coefficient across the 24 correlations was 0.082 and the maximum 0.240, and every confidence interval included zero, including that of the single nominal association. Intervals were obtained by percentile bootstrap with 5000 resamples. Within each instrument, by contrast, subscales were associated with one another, so the absence of cross-instrument association is not attributable to a lack of variance or structure in the measures. The four sensory processing quadrants were uniformly and moderately intercorrelated (ρ = 0.37 to 0.53), whereas the six behavioural subscales formed two clusters, Conduct Problem with Insecure/Anxious (ρ = 0.46) and Hyperactive with Self-Injury/Stereotypic, Self-Isolated/Ritualistic and Overly Sensitive (ρ = 0.18 to 0.40), associations between the two clusters being null to negative (ρ = −0.35 to −0.04). The pattern is therefore one of no detectable association between caregiver-reported sensory processing and caregiver-reported behavioural difficulties, even though both instruments were completed by the same informants (Table S1).

### 3.5. Predictive Value of Sensory Processing Profiles on Sensory-Motivated Behaviours (RQ3)

Across the full sample (*N* = 70), a substantial proportion of children exhibited no SMBs during the observation period. Specifically, 64.3% had no recorded sensory-seeking episodes, and 37.1% had no sensory-avoiding episodes.

#### 3.5.1. Sensory-Seeking Behaviours

In the model for sensory seeking (*n* = 52; 167 sensory-seeking episodes), none of the three predictors was associated with the proportion of episodes assigned that function, and the theoretically expected association with the Sensation Seeking quadrant was not observed (Table 3).

#### 3.5.2. Sensory-Avoidance Behaviours

In the model for sensory avoiding (*n* = 52; 285 sensory-avoiding episodes), none of the three predictors was associated with the proportion of episodes assigned that function (Table 3).

#### 3.5.3. Concordance Across Analytic Strategies

The estimate for adaptive functioning was examined under three strategies (Table S7). Complete-case and multiple-imputation analyses agreed closely for both outcomes, with fractions of missing information highest for adaptive functioning (0.205 and 0.244), as expected given that missingness was confined to the VABS-II. A third strategy replaced the age-referenced v-scale sum with the summed raw score, which is not adjusted for age, and a coefficient appeared for sensory avoiding: −0.00421 (*p* = 0.074) against −0.00017 (*p* = 0.987) under the primary measure, while sensory seeking remained null under both. The two measures are only moderately related (Pearson r = 0.591, Spearman ρ = 0.705) and dispersion parameters were essentially unchanged across specifications, so the difference arises from the predictor rather than from model fit: what the raw score captures is in part age itself, which the primary measure, expressed relative to age norms, removes.

Sensitivity analyses and model diagnostics are reported in Tables S3 and S5: adding age or gender, excluding the four participants beyond the validation range of the Child Sensory Profile-2, and testing linearity on the link scale left every estimate unchanged in direction and magnitude, and collinearity was not a limiting factor (VIF 1.09 to 1.98). These checks were run on the raw-score specification; the quadrant coefficients differ little between the two specifications, so they bear on estimates close to those of the primary model.

## 4. Discussion

This study shifted the level of analysis from caregiver-reported traits to the ecological organisation of behaviour observed in naturalistic contexts, using repeated observations collected through descriptive functional assessment by mobile teams in participants’ everyday environments as part of routine clinical practice. Sensory-motivated behaviours accounted for a small proportion of the episodes recorded, with marked inter-individual variability. The analyses did not detect consistent associations between that proportion and either caregiver-reported sensory responsivity or adaptive functioning, an apparent association with adaptive functioning arising only when the measure is left unadjusted for age. Where structure emerged, it was internal to the observed behaviour: whether the function was seeking or avoiding tracked the form of the episode, systematically and with effects large enough to survive correction for multiple comparisons. Together, these findings locate the informative structure at the level of the episode rather than at the level of the trait.

That episode-level structure takes a simple form. Among episodes assigned a sensory function, avoidance predominated for behaviours directed at the body, whether the child’s own or another person’s, while seeking predominated for behaviours acting upon objects or upon oral intake. The pattern follows from what each behaviour can accomplish: striking, biting or fleeing removes stimulation already present, whereas breaking an object or mouthing a non-food substance generates stimulation that was not there. Topography therefore constrains the sensory consequences a behaviour can produce, and the functional hypotheses recorded by practitioners track that constraint. Clinically, aggressive and self-injurious episodes invite the question of what stimulation the child may be escaping, and destructive, sexualised or eating-related episodes the question of what stimulation the behaviour supplies.

The absence of robust associations between sensory processing profiles and challenging behaviours stands apart from a substantial body of literature documenting links between sensory responsivity differences and maladaptive behaviour in autism and related neurodevelopmental conditions (Baranek et al., 2006; Chen et al., 2024; Dellapiazza et al., 2020; Fabbri-Destro et al., 2022; Griffin et al., 2022; Lane et al., 2010; Leekam et al., 2011). Much of that literature relies on caregiver-report questionnaires, which capture relatively stable, trait-like sensory predispositions and global behavioural tendencies rather than the functional organisation of behaviour as it unfolds in context. This reliance on proxy reporting is particularly limiting in populations with intellectual disability or limited verbal communication and provides restricted insight into contextual contingencies. Discrepancies between caregiver report and observed behaviour have been documented in minimally verbal children with autism (Lane et al., 2010; Tager-Flusberg et al., 2017). Because the present assessments were themselves conducted within participants’ everyday environments, including the home, the divergence from caregiver questionnaires is unlikely to reflect a simple difference in setting (for example, home versus clinic); it more plausibly arises from the contrast between global, trait-level proxy report and episodic, function-level direct observation.

Characteristics of the present sample may also contribute to this divergence. Participants were referred to specialised services because of severe, persistent challenging behaviours, a population underrepresented in research on sensory–behaviour relationships. Studies of sensory contributions in comparable populations have typically isolated sensory factors through experimental or intervention-based frameworks, including functional analyses (Patel et al., 2000), modality-specific investigations of tactile or multisensory reactivity (Marco et al., 2011; Gunderson et al., 2025), and sensory-based treatment trials following functional assessment (Devlin et al., 2009, 2011; Reichow et al., 2009), which presuppose an isolability that may not hold in ecologically complex contexts. This study instead examined behaviour as it unfolds in naturalistic settings, where challenging behaviours appear as multidetermined patterns in which sensory factors are intertwined with adaptive functioning, regulatory capacities, arousal, pain sensitivity, and contextual contingencies, and where sensory contributions may not emerge as strong predictors of global behavioural frequency when assessed indirectly. This is consistent with studies showing that many behaviours presumed to be sensory-driven are in fact maintained by non-sensory contingencies (Iwata et al., 1994; Piazza et al., 2000; Devlin et al., 2009, 2011).

Sensory-motivated behaviours are best understood as a coding framework for describing challenging behaviour in ecological terms. They are neither a behavioural phenotype nor a direct manifestation of sensory processing profiles: profiles index stable predispositions, whereas the category captures context-dependent expressions inferred from the antecedents and consequences recorded in the setting. Consistent with that distinction, no caregiver-reported quadrant tracked its functionally corresponding subtype, and none was associated with the proportion of episodes assigned a sensory function. The findings are compatible with models in which sensory characteristics operate through regulatory and adaptive mechanisms rather than directly (Dellapiazza et al., 2020; Kanne & Mazurek, 2011).

The low proportion of episodes classified as sensory-motivated further challenges the assumption that sensory processing differences constitute a primary or ubiquitous driver of challenging behaviour, an assumption largely built on questionnaire-based indices of behavioural frequency or severity rather than on functional classification of individual episodes (Aman et al., 1996; Lecavalier et al., 2004). A second null result bears on this reading: the four sensory quadrants were no more strongly associated with caregiver-reported behavioural difficulties than with observed sensory functions, since across 24 correlations the median absolute coefficient was 0.082 and every interval included zero, even though both instruments were completed by the same informants. The divergence between the questionnaire and the observational level therefore cannot be attributed to the change of informant alone. Nor can it be attributed to a sample in which sensory concerns were peripheral: because the Sensory Profile-2 was administered on clinical indication, the children analysed here are those for whom a clinician judged sensory features salient enough to justify formal assessment, so the sample was if anything weighted towards children whose difficulties were expected to be sensory in nature. The discordance between clinically anticipated sensory relevance and observed functional attribution is thus a substantive finding in its own right, and one that questionnaire-based designs are not positioned to detect.

Marked inter-individual variability in the proportion of episodes assigned a sensory function further suggests that sensory factors may exert episodic, state-dependent influences rather than acting as stable determinants of behaviour. Sensory-related challenges may contribute to behavioural destabilisation under specific conditions, such as heightened arousal, fatigue, pain, or reduced regulatory resources, without shaping baseline behavioural patterns. This interpretation aligns with work on multisensory hypersensitivity, hyperarousal, and sensory modulation difficulties, which emphasises state-dependent effects rather than stable behavioural drivers (Baranek et al., 2006; Marco et al., 2011). A preliminary comparison, unplanned and resting on 16 girls, found a difference by gender that was confined to sensory avoiding, with no counterpart for sensory seeking. Its form is notable: none of the 16 girls was without an episode of sensory avoiding, whereas 26 of the 54 boys were, so the difference concerns whether such episodes occurred at all rather than how often they occurred among children who displayed them. The effect size for sensory avoiding was large and precisely bounded (rank-biserial −0.616, 95% CI −0.794 to −0.407), and the association held in a model including age. These data cannot distinguish between differences in behavioural expression, in environmental demands, and in what practitioners recorded (Griffin et al., 2022).

Methodologically, repeated naturalistic observations collected through descriptive functional assessment give access to the contingencies under which behaviour occurs, which caregiver questionnaires index only indirectly. Adaptive functioning was itself assessed through a caregiver-based instrument, the VABS-II, and showed no association with the proportion of sensory episodes. The comparison between direct observation and caregiver report therefore rests on the constructs each captures rather than on a difference in predictive performance, with caregiver instruments indexing stable skills and predispositions and functional observation characterising the contingencies under which behaviours occur.

Clinically, these findings caution against over-attributing behaviours to sensory processing differences based solely on questionnaire profiles. Identifying a sensory contribution does not, in itself, imply that sensory-based interventions are indicated or sufficient. This conclusion is consistent with comparative intervention studies demonstrating stronger and more reliable effects of function-based behavioural approaches than non-individualised sensory interventions for challenging behaviour (Devlin et al., 2009, 2011). What the present findings support is that questionnaire-derived sensory profiles and function-level observation carry different information, so that a sensory-motivated behaviour is best used as a starting point for formulation and hypothesis generation rather than as a category prescribing an intervention.

### 4.1. Strengths and Limitations

A major strength of this study is its ecological approach, relying on repeated naturalistic observations conducted through descriptive functional assessment in participants’ everyday environments (home, school, and day-care settings) rather than in clinic-based assessments, which gave direct access to the organisation of challenging behaviours as they occurred rather than to trait-level inferences derived from questionnaires. A second is the integration of multiple methodological levels, combining standardised caregiver-report instruments (including the Sensory Profile-2 and the NCBRF) with functional behavioural data derived from routine clinical practice, so that sensory predispositions could be examined alongside their behavioural expression rather than conflated with it. This secondary use of routinely collected clinical data also reduces participant burden and makes fuller use of existing clinical records. Finally, the study focused on a clinically underrepresented population, children referred to specialised services as a result of severe and persistent challenging behaviours, thereby addressing an important gap in the sensory processing literature.

Several limitations bear on how these findings should be read. The functional attribution of behaviours as sensory-motivated rests on descriptive rather than experimental functional analyses and remains inferential: SMBs are clinically informed hypotheses about the function of an episode, not definitive classifications. The construct validity of the category and its correspondence with experimental functional analysis remain to be established, and both would be required before it could be treated as a measurement category rather than as a framework supporting clinical reasoning. Only linear associations were tested; a quadratic term for adaptive functioning did not improve model fit, but no comparable test was conducted for the sensory quadrants. The coding grid is the service’s own. Four of its eight categories correspond to topographies routinely distinguished in the challenging-behaviour literature; the other four were built for the programme, and one of them, behavioural crises, aggregates several topographies within a single record. The class-by-function results therefore describe this grid, and replication requires one constructed independently. Two estimates in those models rest on thin cells: that for eating-related behaviours on a single episode, so only its direction should be retained, and the stereotyped category comprises a single item, stereotyped auditory behaviour (76 episodes, six children), motor, visual and tactile forms being absent from the coding grid.

The analytic sample is not a random subset of eligible clients. The Sensory Profile-2 was administered when a clinician judged a sensory evaluation to be indicated, further protocols were lost through archiving and encoding practices, and return of a caregiver-completed questionnaire may itself have been associated with family circumstances; the sample is therefore enriched in children for whom sensory features were judged clinically salient, and the characteristics of excluded eligible clients could not be recovered. Four participants exceeded the upper validation limit of the Child Sensory Profile-2, whose norms extend to 14 years 11 months, although excluding them left every estimate unchanged in direction and magnitude in the raw-score specification, on which that check was run (Table S5). Clinical heterogeneity and unbalanced subgroup sizes limited power for the exploratory comparisons, particularly by age, diagnostic group and gender, and replication in larger and more balanced samples will be necessary. Diagnoses were established outside the service and prior to admission, with the instruments and criteria of the referring services undocumented, so diagnostic practice may have varied across services and over the recruitment period. Severity of intellectual disability was undocumented for close to half the sample and no cognitive or developmental quotient data were available, so developmental level could be characterised only through adaptive functioning, itself constrained: the Adaptive Behaviour Composite was at the instrument floor for 44 of the 50 participants for whom it could be computed, and the Communication and Socialisation standard scores for 44 and 45 of 52. That last point is a mismatch between the range of the instrument and the severity of this population, and it bears on any study using adaptive behaviour as a continuous variable in comparable samples.

Reliability was established at one level only. Blind independent recoding gives κ = 0.86 (95% CI 0.82 to 0.90; Section 3.1) for the research coding step, so that operation is reproducible. The practitioners’ original functional hypotheses, the delimitation of episodes and their assignment to behavioural categories were all produced in situ during routine care between 2018 and 2025 by teams whose composition changed over the period, and cannot be reassessed; systematic differences between teams and drift over the recruitment period therefore remain possible, and classification error of unknown magnitude bears on the primary outcome.

The disagreements locate where the coding is least secure. Agreement was near-perfect for sensory avoiding and lower for sensory seeking, and most disagreements in that category concerned hypotheses designating self-stimulation, where the assignment turns on whether the recorded antecedent refers to sensory stimulation. Sensory-seeking classifications are therefore less secure than sensory-avoiding ones. The presence of an observer may also have altered the behaviour recorded, particularly early in the observation period, and episodes were aggregated across the three-month window without modelling within-person change.

Three qualifications concern the data and the design. VABS-II data were missing for 18 participants; missingness was unrelated to the outcome and concentrated in the ASD group, and complete-case and multiple-imputation results agree closely (Section 3.5.3), although dependence on the unobserved values themselves cannot be excluded. The raw-score estimate for adaptive functioning reported as a sensitivity analysis is itself fragile: removing any single participant moves its *p*-value between 0.035 and 0.204, though never its direction, which reflects the small number of participants at the extremes of the predictor. The number of participants relative to the number of predictors also remains modest, which is why models were restricted to three prespecified predictors and the model containing all four quadrants is reported as exploratory. Finally, the study compared neither assessment approaches nor interventions, so the clinical implications drawn above are hypotheses to be tested.

### 4.2. Perspectives

Future research should integrate ecological observation with experimental and longitudinal designs to clarify the conditions under which sensory factors contribute to the organisation and maintenance of challenging behaviours; combining descriptive functional assessment with targeted experimental manipulations may help disentangle sensory contributions from overlapping processes such as emotional regulation, arousal, and pain sensitivity. State-dependent and context-sensitive models deserve particular attention in clinically complex populations, examining how sensory challenges interact with fluctuating regulatory resources, environmental demands, and adaptive skills over time rather than treating sensory differences as stable behavioural drivers. In populations with severe intellectual disability or limited verbal communication, this will require multimethod designs combining caregiver-report instruments, direct behavioural observation, and psychophysiological measures.

One source of information remains conspicuously absent from this literature, and from the present study: the account of the child or adolescent. Functional assessment protocols have long included interview formats that solicit information directly from the individual whose behaviour is under assessment, notably the Student-Assisted Functional Assessment Interview (Kern et al., 1994) and the student-directed interview included in O’Neill et al. (2015). Where such formats have been evaluated, agreement between the young person’s account and that of their teachers has been substantial for the perceived causes and functions of behaviour, though not for the support strategies subsequently proposed (Reed et al., 1997). No such interview was conducted in the records analysed here, and the level of adaptive communication in this sample would have limited its feasibility for many participants. The approach nonetheless deserves systematic attention in populations where it is possible, since the individual’s own account may revise a functional hypothesis rather than merely corroborate it, and this is particularly relevant to sensory motivation, where the salient event is an internal experience that an observer can only infer. Extending it to children with limited speech would require adapted formats, including augmentative and alternative communication supports and visually structured interviews, whose validity for functional attribution remains to be established.

## 5. Conclusions

This study re-examined the role attributed to sensory processing differences in challenging behaviour associated with ASD and intellectual disability by working at the level of the observed episode rather than the caregiver-reported trait. Under the service’s descriptive procedures, few episodes were classified as sensory-motivated, and those that were proved unevenly distributed across children and across behavioural forms; because only explicitly designated sensory functions were retained, that figure indexes how often practitioners named a sensory function rather than how often behaviour served one.

The findings point to a discordance between levels of measurement. No consistent correspondence emerged between what caregivers report about a child’s sensory responsivity and the sensory functions attributed to that child’s behaviour in context, nor between the two caregiver-report instruments, although both were completed by the same informants. Where structure was found, it was internal to the observed behaviour, seeking and avoiding tracking the form of the episode, body-directed behaviours being associated with avoidance and object- or intake-directed behaviours with seeking. For practice, a caregiver-reported sensory profile is therefore not a substitute for functional assessment of the behaviour itself; for research, the episode is the level at which sensory function becomes legible in clinical records.

## Figures and Tables

**Table 1:** Demographic and clinical characteristics of participants (*N* = 70). Age is reported in completed years; the exact mean age in decimal years is 10.00 (*SD* 3.09). Sensory Profile-2 and NCBRF values are raw scores. NCBRF statistics are based on 69 participants, one participant’s subscale values having been imputed at an earlier stage without documentation. VABS-II statistics are based on the 52 participants with a completed assessment. Floor refers to the lowest obtainable standard score of 20.

N	70
Age in years, completed years	9.6 (3.02); median 10 [7–12]; range 3–16
Gender m/f (%)	54 (77.14%)/16 (22.86%)
**Diagnosis, *N* (%)**	
ASD and co-occurring intellectual disability	60 (85.71%)
Intellectual disability alone	10 (14.29%)
**Severity of Intellectual Disabilities, *N* (%)**	
Mild	1 (1.43%)
Moderate	11 (15.71%)
Severe (*n* = 22)/profound (*n* = 3)	25 (35.71%)
Unspecified	33 (47.15%)
**Medication, *N* (%)**	
Polypharmacy	20 (28.57%)
Single medication	28 (40.00%)
No medication reported	22 (31.43%)
**Dunn’s Sensory Profile-2, raw scores:** ***M* (*SD*); median [IQR]; range**	
Low Registration	46.10 (12.84); 45.5 [37.00–55.50]; 21–79
Sensation Avoiding	49.53 (12.63); 50.0 [43.25–57.00]; 8–83
Sensation Seeking	49.34 (14.87); 51.5 [38.25–58.75]; 19–98
Sensory Sensitivity	44.17 (13.08); 43.0 [36.50–52.50]; 10–80
**Nisonger Child Behaviour Rating Form, raw scores (*n* = 69): *M* (*SD*); median [IQR]; range**	
Conduct Problem	7.86 (2.99); 8 [6–10]; 2–16
Insecure/Anxious	4.13 (2.65); 4 [2–6]; 0–13
Hyperactive	19.03 (8.82); 18 [14–23]; 0–40
Self-Injury/Stereotypic	6.90 (5.48); 5 [3–10]; 0–35
Self-Isolated/Ritualistic	17.10 (6.19); 17 [12–22]; 4–27
Overly Sensitive	5.46 (3.91); 6 [2–7]; 0–17
Compliance/Calm	7.46 (4.43); 7 [4–10]; 0–17
Adaptive Social	8.04 (3.32); 7 [6–11]; 2–17
**Vineland Adaptive Behaviour Scales-II, raw scores (*n* = 52): *M* (*SD*); median [IQR]; range**	
Communication	54.31 (35.60); 42 [28.75–72.00]; 13–166
Daily living	48.27 (24.40); 45 [31.25–64.00]; 10–111
Socialisation	44.81 (24.05); 36 [29.75–57.00]; 12–149
Total summed raw score	147.38 (75.19); 119 [95.00–186.25]; 54–347
**Vineland Adaptive Behaviour Scales-II, norm-referenced (*n* = 52): median [IQR]; range**	
Communication standard score	20 [20–20]; 20–81 (44 of 52 at floor)
Daily living standard score	20 [20–31]; 20–70 (30 of 52 at floor)
Socialisation standard score	20 [20–20]; 20–82 (45 of 52 at floor)
Adaptive Behaviour Composite (*n* = 50)	20 [20–20]; 20–48 (45 of 50 at floor)
Sum of the nine v-scale scores	20 [14.75–29]; 9–75
Number of analysed behaviour episodes (*M*, *SD*)	108.00 (82.96); 77 [44.25–154.50]; 9–362

Note. *M* = mean; *SD* = standard deviation. Sensory Profile-2 and Nisonger Child Behaviour Rating Form values correspond to raw scores. Vineland Adaptive Behaviour Scales-II values represent the sum of raw scores for each adaptive domain. VABS-II data were unavailable for 18 participants; complete-case analyses are primary and multiple imputation is reported as a sensitivity analysis. Medication data were extracted from routine clinical records and correspond to secondary information reported by caregivers and healthcare professionals. Polypharmacy was defined as the regular use of two or more psychotropic medications belonging to distinct pharmacological classes, including typical and atypical antipsychotics, methylphenidate, antidepressants, anticonvulsants/antiepileptics and benzodiazepines. Non-psychotropic medications (e.g., analgesics, anti-reflux treatments) were not considered. The number of analysed behaviour episodes corresponds to the number of distinct challenging behaviour episodes that were subjected to descriptive functional analysis and subsequently recorded in the study database for each participant.

**Table 2:** Distribution of behavioural categories according to inferred sensory function.

Behavioural Categories	Non-Sensory Motivated Behaviour (nSMBs)	Sensory-Avoiding SMBs	Sensory-Seeking SMBs	Total
Self-injurious behaviours	621 (8.21%)	54 (0.71%)	15 (0.20%)	690 (9.13%)
Aggression towards others	1568 (20.74%)	106 (1.40%)	11 (0.15%)	1685 (22.29%)
Socially inappropriate behaviours	3124 (41.32%)	149 (1.97%)	67 (0.89%)	3340 (44.18%)
Eating-related behaviours	254 (3.36%)	1 (0.01%)	24 (0.32%)	279 (3.69%)
Sexualised behaviours	105 (1.39%)	1 (0.01%)	35 (0.46%)	141 (1.87%)
Destruction of materials	1051 (13.90%)	21 (0.28%)	74 (0.98%)	1146 (15.16%)
Stereotyped behaviours	72 (0.95%)	3 (0.04%)	1 (0.01%)	76 (1.01%)
Behavioural crises or meltdowns	200 (2.65%)	3 (0.04%)	0 (0%)	203 (2.69%)
**Total**	**6995** **(92.53%)**	**338** **(4.47%)**	**227 (3.00%)**	**7560** **(100%)**

Note. Values represent observed frequencies of behaviours classified into eight behavioural classes and categorised according to inferred sensory function (non-sensory motivated behaviour, sensory-avoiding motivated behaviour, or sensory-seeking motivated behaviour) based on descriptive functional assessments conducted in naturalistic settings. Percentages are reported in regard to the total number of behaviours (*n* = 7560). Bold type indicates the header row and the row and column totals.

**Table 3:** Beta-binomial models of the proportion of episodes assigned each sensory function (complete cases, *n* = 52), with adaptive functioning measured as the sum of age-referenced v-scale scores. Odds ratios are expressed per 10-point increment of the predictor.

Outcome and Predictor	OR per 10 Points	95% CI	*p*
Sensory seeking (167 episodes; dispersion 5.22)			
Sensation Seeking	1.162	0.771 to 1.751	0.473
Low Registration	0.961	0.618 to 1.494	0.861
Adaptive functioning (VABS-II v-scale sum)	0.871	0.609 to 1.247	0.451
Sensory avoiding (285 episodes; dispersion 15.83)			
Sensation Avoiding	1.093	0.798 to 1.496	0.580
Sensory Sensitivity	0.854	0.628 to 1.161	0.314
Adaptive functioning (VABS-II v-scale sum)	0.998	0.813 to 1.226	0.987

Note. The dispersion parameter is a precision, so lower values indicate stronger overdispersion. The v-scale sum ranges from 9 to 75 in this sample, so a 10-point increment spans a larger share of the observed range than for the Sensory Profile-2 quadrants. Predictors were prespecified from the directional hypotheses; the model containing all four quadrants is reported in Table S2.

## Data Availability

The data supporting the findings of this study are available from the corresponding author upon reasonable request. The data are not publicly available due to privacy and ethical restrictions relating to sensitive clinical data involving children with ASD and/or intellectual disability.

## Supplementary Materials

The following supporting information can be downloaded at: https://www.mdpi.com/article/10.3390/bs16091561/s1, Table S1. Full matrix of the 24 Spearman correlations between Sensory Profile-2 quadrants and NCBRF maladaptive subscales (*n* = 69). Table S2. Exploratory beta-binomial models containing all four sensory quadrants together with adaptive functioning, with variance inflation factors. Table S3. Model diagnostics: variance inflation factors, Pearson dispersion, binomial versus beta-binomial fit, linearity on the link scale, and single-participant deletion influence analysis. Table S4. Multiple imputation: imputation model, number of datasets and iterations, convergence checks, fractions of missing information, and preservation of the between-domain correlation structure. Table S5. Sensitivity analyses adding age and gender separately, and excluding the four participants beyond the validation range of the Child Sensory Profile-2 (*n* = 48). Table S6. VABS-II norm-referenced scores by domain and subdomain, including v-scale sums, standard scores, and the Adaptive Behaviour Composite, with the derivation of each metric, floor distributions per metric, and summation diagnostics. Table S7. Coefficient for adaptive functioning across analytic strategies: complete cases, multiple imputation, and the unadjusted summed raw score, with standard errors and fractions of missing information. Table S8. Behavioural category definitions and function coding rules: operational definitions of the eight behavioural categories, unit of analysis and a worked example of the crisis unit, the two levels of decision, the rule restricting sensory coding to explicitly designated sensory functions, and the additional rule applied to self-stimulation. Table S9. Mixed-effects logistic models of the inferred sensory function by behavioural class (random intercept for participant; reference class: socially inappropriate behaviours). Panel A: whether an episode was assigned a sensory function (7560 episodes, 70 participants). Panel B: whether that function was avoiding rather than seeking, among the 565 sensory episodes.
