# Supplementary material for: Sensory Processing Profiles and Observed Behaviour: An Ecological, Function-Based Study of Challenging Behaviour in a Clinically Referred Sample of Children with Autism Spectrum Disorder and/or Intellectual Disability
Source: Behav Sci (Basel). 2026 Sep 3;16(9):1561. doi: 10.3390/bs16091561 (PMC13603829; doi:10.3390/bs16091561)
Supplement: Supplementary file 1 [file behavsci-16-01561-s001.zip › behavsci-4445470-supplementary.pdf]

Supplementary Materials

Sensory Processing Profiles and Observed Behaviour: An Ecological, Function-Based Study of Challenging Behaviour in a Clinically Referred Sample of Children with Autism Spectrum Disorder and/or Intellectual Disability

All analyses were conducted in R 4.3.3 using glmmTMB 1.1.8 for the beta-binomial and mixed-effects models and mice 3.16.0 for multiple imputation, with a fixed random seed (20260810). Sample sizes differ across analyses and are stated for each: 70 participants for analyses not involving the VABS-II, 52 for complete-case analyses involving it, 69 for analyses involving the NCBRF, and 48 for the sensitivity analysis excluding participants beyond the validation range of the Child Sensory Profile-2. Adaptive functioning is measured as the sum of age-referenced v-scale scores in the primary models, reported in Table 3 of the manuscript. The diagnostics and sensitivity analyses in Tables S2 to S5 were computed on the summed raw score, which the manuscript reports as a sensitivity analysis; Table S7 compares the two measures directly.

Table S1. Full matrix of the 24 Spearman correlations between Sensory Profile-2 quadrants and NCBRF maladaptive subscales (n = 69).

Sensory Profile-2 quadrant	NCBRF maladaptive subscale	ρ	95% CI	p	p (Holm)	p (BH)
Sensation Seeking	Conduct Problem	−0.011	−0.261 to 0.246	.928	1.000	.993
Sensation Seeking	Insecure/Anxious	0.001	−0.244 to 0.247	.993	1.000	.993
Sensation Seeking	Hyperactive	−0.091	−0.327 to 0.163	.458	1.000	.926
Sensation Seeking	Self-Injury/Stereotypic	−0.127	−0.374 to 0.124	.300	1.000	.926
Sensation Seeking	Self-Isolated/Ritualistic	0.082	−0.155 to 0.324	.502	1.000	.926
Sensation Seeking	Overly Sensitive	0.163	−0.083 to 0.392	.181	1.000	.926
Sensation Avoiding	Conduct Problem	0.182	−0.057 to 0.405	.134	1.000	.926
Sensation Avoiding	Insecure/Anxious	0.047	−0.202 to 0.289	.701	1.000	.993
Sensation Avoiding	Hyperactive	−0.014	−0.242 to 0.215	.910	1.000	.993
Sensation Avoiding	Self-Injury/Stereotypic	0.240	−0.007 to 0.457	.047	1.000	.926
Sensation Avoiding	Self-Isolated/Ritualistic	0.121	−0.109 to 0.335	.320	1.000	.926
Sensation Avoiding	Overly Sensitive	−0.002	−0.242 to 0.241	.986	1.000	.993
Sensory Sensitivity	Conduct Problem	−0.062	−0.281 to 0.166	.612	1.000	.993
Sensory Sensitivity	Insecure/Anxious	−0.029	−0.272 to 0.221	.814	1.000	.993
Sensory Sensitivity	Hyperactive	−0.195	−0.420 to 0.048	.108	1.000	.926
Sensory Sensitivity	Self-Injury/Stereotypic	0.033	−0.220 to 0.272	.788	1.000	.993
Sensory Sensitivity	Self-Isolated/Ritualistic	−0.094	−0.332 to 0.151	.444	1.000	.926
Sensory Sensitivity	Overly Sensitive	−0.051	−0.308 to 0.202	.675	1.000	.993
Low Registration	Conduct Problem	0.119	−0.106 to 0.345	.331	1.000	.926
Low Registration	Insecure/Anxious	0.119	−0.149 to 0.373	.331	1.000	.926
Low Registration	Hyperactive	0.020	−0.234 to 0.277	.871	1.000	.993
Low Registration	Self-Injury/Stereotypic	0.034	−0.199 to 0.258	.782	1.000	.993
Low Registration	Self-Isolated/Ritualistic	0.083	−0.164 to 0.322	.497	1.000	.926
Low Registration	Overly Sensitive	0.115	−0.132 to 0.355	.349	1.000	.926

Note. Confidence intervals were obtained by percentile bootstrap with 5,000 resamples. Holm and Benjamini–Hochberg adjustments were applied across the 24 tests. One participant was excluded from all NCBRF analyses because the eight subscale values had been imputed at an earlier stage without documentation. The median absolute coefficient is 0.082 and the maximum 0.240; every confidence interval includes zero.

Table S2. Exploratory beta-binomial models containing all four sensory quadrants together with adaptive functioning (complete cases, n = 52).

Predictor	OR per 10 points	95% CI	p	VIF
Sensory seeking (n = 52; 167 episodes; dispersion 5.40; AIC 183.3)				
Sensation Seeking	1.289	0.802 to 2.070	.295	1.899
Sensation Avoiding	0.981	0.633 to 1.518	.930	1.735
Sensory Sensitivity	0.745	0.424 to 1.309	.306	1.981
Low Registration	0.980	0.626 to 1.535	.931	1.564
Adaptive functioning (VABS-II raw total)	0.986	0.918 to 1.059	.698	1.413
Sensory avoiding (n = 52; 285 episodes; dispersion 18.70; AIC 268.7)				
Sensation Seeking	1.383	1.017 to 1.879	.038	1.899
Sensation Avoiding	1.147	0.841 to 1.563	.387	1.735
Sensory Sensitivity	0.742	0.523 to 1.053	.095	1.981
Low Registration	0.753	0.552 to 1.026	.072	1.564
Adaptive functioning (VABS-II raw total)	0.976	0.930 to 1.024	.325	1.413

Note. Odds ratios are expressed per 10-point increment of the predictor. These models were not prespecified and are reported for completeness. The nominal association between Sensation Seeking and sensory avoiding (p = .038) appears in this model only, was not hypothesised in that direction, and does not survive correction for five predictors; it is reported without interpretation. VIF = variance inflation factor. The dispersion parameter is a precision, so lower values indicate stronger overdispersion.

Table S3. Model diagnostics for the beta-binomial models.

Diagnostic	Value	Reference
Multicollinearity (variance inflation factors)		
Sensory-seeking model (3 predictors)	1.213 to 1.572	Threshold of concern: 5
Sensory-avoiding model (3 predictors)	1.090 to 1.667	Threshold of concern: 5
Exploratory model (5 predictors)	1.413 to 1.981	Threshold of concern: 5
Overdispersion and model fit		
Pearson dispersion, binomial model, sensory seeking	21.50 (df = 48)	1.00 expected under the binomial
Pearson dispersion, binomial model, sensory avoiding	4.62 (df = 48)	1.00 expected under the binomial
AIC, sensory seeking: binomial vs. beta-binomial	543.5 vs. 180.7	ΔAIC = 362.8
AIC, sensory avoiding: binomial vs. beta-binomial	360.4 vs. 269.8	ΔAIC = 90.6
Linearity on the link scale (quadratic term for adaptive functioning)		
Sensory seeking	$\chi^2(1) = 0.84$, p = .359	Linearity supported
Sensory avoiding	$\chi^2(1) = 0.01$, p = .930	Linearity supported
Influence: leave-one-participant-out (52 refits per model)		
Sensory seeking, coefficient for adaptive functioning	−0.00445 to −0.00136	Full-sample estimate −0.00214
Sensory seeking, p-value	.285 to .706	Full-sample p = .544
Sensory avoiding, coefficient for adaptive functioning	−0.00551 to −0.00308	Full-sample estimate −0.00421
Sensory avoiding, p-value	.035 to .204	Full-sample p = .074
Maximum absolute dfbeta across both models	0.65	Threshold of concern: 1

Note. Variance inflation factors were computed from the auxiliary regressions among predictors. Pearson dispersion is the sum of squared Pearson residuals divided by the residual degrees of freedom under a binomial model; a value of 1 indicates no overdispersion. Linearity was assessed by adding a quadratic term for adaptive functioning and comparing nested models. The influence analysis refits each model 52 times, omitting one participant at a time. The five most influential participants lie in the upper part of the adaptive functioning distribution, four having raw totals between 245 and 347 against a sample median of 119. No value was aberrant, the three domains being mutually consistent within each of these participants, and no participant was excluded.

Table S4. Multiple imputation of the Vineland-II: imputation model, diagnostics, and rationale for the primary analysis.

**Element**	**Specification**
Procedure	
Software	R 4.3.3, mice 3.16.0
Missingness	18 of 70 participants (25.7%), all nine VABS-II subdomains absent
Variables imputed	The three domain raw totals, imputed separately
Total score	Recomputed by summation (passive imputation), ensuring internal consistency
Imputation method	Predictive mean matching, five donors
Predictors	Exact age in months, gender, diagnostic group, the four Sensory Profile-2 quadrants, log of the total number of episodes, empirical logit of the proportion of sensory episodes, and the two remaining VABS-II domains
Number of imputed datasets	50
Iterations	20
Random seed	20260810
Pooling	Rubin's rules, with Barnard–Rubin degrees of freedom
Missingness assumption	Missing at random, conditional on diagnostic group
Construction checks	
Imputed values outside the observed range	0
Non-integer imputed values	0
Discrepancy between the total and the sum of the three domains	0
Convergence	Assessed by inspection of trace plots of the means and standard deviations of the imputed values for the three domain raw totals: the 50 chains overlapped without separation and showed no systematic drift over the final iterations (plots not shown; reproducible from the fixed random seed)
Preservation of the between-domain correlation structure	
Observed ($n = 52$)	r(Com, DLS) = 0.695; r(Com, Soc) = 0.756; r(DLS, Soc) = 0.605
Present imputation, imputed participants ($n = 18$)	0.553; 0.654; 0.545
Earlier single imputation, imputed participants ($n = 18$)	−0.011; 0.521; 0.471
Fractions of missing information (pooled models, n = 70)	
Sensation Seeking / Sensation Avoiding	0.051 / 0.060
Low Registration / Sensory Sensitivity	0.037 / 0.069
Adaptive functioning, sensory seeking / sensory avoiding	0.205 / 0.244
Rationale for retaining complete cases as the primary analysis	
Association between missingness and the outcome	Rank-biserial +0.112, $p = .485$
Association between missingness and diagnostic group	18 of 60 in the ASD group vs. 0 of 10 in the ID-only group, Fisher's exact $p = .054$
Adjusted R^2 of the imputation model for the summed raw total	0.187
Reduction in uncertainty about a missing value	10% (residual SD 67.8 vs. marginal SD 75.2)

Note. Predictive mean matching draws each imputed value from participants with a similar predicted score, so imputed values are integer, within the observed range, and drawn from the same clinical population. The outcome was included in the imputation model on the scale of the analysis model; omitting it would attenuate the estimated associations towards the null. Because the missingness is unrelated to the outcome and the incomplete variable is a predictor rather than the outcome, complete-case analysis remains unbiased, which together with the limited information available for imputation motivates its retention as the primary analysis. Dependence on the unobserved values themselves cannot be excluded and is discussed in the Limitations.

Table S5. Sensitivity analyses: adding age and gender separately, and excluding the four participants beyond the validation range of the Child Sensory Profile-2.

Predictor	Raw-score model	Adding age	Adding gender	Excluding 4 participants
Sensory seeking				
Sensation Seeking	1.125 (.601)	1.139 (.561)	1.194 (.436)	1.156 (.533)
Low Registration	0.965 (.874)	0.967 (.882)	0.915 (.698)	0.957 (.850)
Adaptive functioning	0.979 (.544)	0.978 (.538)	0.971 (.397)	0.981 (.576)
Age (per year)	—	1.043 (.564)	—	—
Gender (girls vs. boys)	—	—	0.491 (.238)	—
n / episodes	52 / 167	52 / 167	52 / 167	48 / 164
Sensory avoiding				
Sensation Avoiding	1.169 (.307)	1.178 (.292)	1.169 (.304)	1.173 (.298)
Sensory Sensitivity	0.828 (.226)	0.795 (.141)	0.802 (.141)	0.802 (.163)
Adaptive functioning	0.959 (.074)	0.961 (.092)	0.968 (.187)	0.962 (.101)
Age (per year)	—	0.937 (.187)	—	—
Gender (girls vs. boys)	—	—	2.176 (.009)	—
n / episodes	52 / 285	52 / 285	52 / 285	48 / 269

Note. Values are odds ratios per 10-point increment of the predictor, with p-values in parentheses; age is expressed per year and gender as girls relative to boys. Confounders were added one at a time as robustness checks rather than as a combined adjustment model. The Child Sensory Profile-2 is validated from 3 to 14 years; four participants aged 15 years 10 months to 16 years 11 months exceeded this range by one to three years. Because the Sensory Profile-2 quadrants enter the models as raw scores, no norm-referenced interpretation of that instrument was affected. No estimate changed in direction, and none changed materially in magnitude. Confidence intervals for the two confounders, omitted from the table for legibility, are as follows: for age, 1.043, 95% CI 0.905 to 1.202, in the sensory-seeking model and 0.937, 95% CI 0.852 to 1.032, in the sensory-avoiding model; for gender, 0.491, 95% CI 0.150 to 1.603, and 2.176, 95% CI 1.210 to 3.914, respectively. Confidence intervals for the prespecified predictors are given in Table 3 of the manuscript.

Table S6. Vineland-II scoring metrics: derivation, distribution, floor effects, and summation diagnostics (n = 52 unless otherwise stated).

Metric	n / input	Median [IQR] / statistic	Range / correlations	At floor
Derivation of each metric				
Raw subdomain score	—	Sum of item scores; not age-referenced	—	—
v-scale score	Raw subdomain score + exact age	Age-referenced; M = 15, SD = 3; range 1 to 24	—	—
Domain standard score	Sum of the three v-scale scores of the domain, converted via the normative table	M = 100, SD = 15; floor value 20	—	—
Adaptive Behaviour Composite	Sum of the domain standard scores, converted via the normative table (three domains from age 7; four, including Motor, below age 7)	M = 100, SD = 15; floor value 20	—	—
Distribution of the norm-referenced metrics				
Standard score, Communication	52	20 [20 to 20]	20 to 81	44 (85%)
Standard score, Daily Living Skills	52	20 [20 to 31]	20 to 70	30 (58%)
Standard score, Socialisation	52	20 [20 to 20]	20 to 82	45 (87%)
Adaptive Behaviour Composite	50	20 [20 to 20]	20 to 48	44 (88%)
v-scale sum, Communication	52	5 [3 to 10]	3 to 38	20 (38%)

Metric	n / input	Median [IQR] / statistic	Range / correlations	At floor
v-scale sum, Daily Living Skills	52	10 [7 to 15]	3 to 32	1 (2%)
v-scale sum, Socialisation	52	3 [3 to 5]	3 to 37	32 (62%)
v-scale sum, nine subdomains	52	20 [14.75 to 29]	9 to 75	1 (2%)
v-scale scores by subdomain (floor value = 1)				
Receptive	52	1 [1 to 5]	1 to 11	29 (56%)
Expressive	52	1 [1 to 1]	1 to 17	47 (90%)
Written	52	1 [1 to 3]	1 to 15	35 (67%)
Personal	52	1 [1 to 1]	1 to 10	45 (87%)
Domestic	52	8 [5 to 9]	1 to 13	1 (2%)
Community	52	1 [1 to 1]	1 to 11	39 (75%)
Interpersonal Relationships	52	1 [1 to 1]	1 to 20	39 (75%)
Play and Leisure Time	52	1 [1 to 1]	1 to 7	47 (90%)
Coping Skills	52	1 [1 to 2]	1 to 13	34 (65%)
Summation diagnostics: internal consistency of the three domains				
Raw domain totals	52	α = 0.852; PC1 = 79%	r = 0.695 / 0.756 / 0.605	—
v-scale sums	52	α = 0.756; PC1 = 68%	r = 0.608 / 0.450 / 0.491	—
Domain standard scores	52	α = 0.664; PC1 = 60%	r = 0.541 / 0.204 / 0.449	—

Note. The Adaptive Behaviour Composite could not be computed for two participants under 7 years of age for whom the Motor domain, which enters the composite below that age, was not administered. Correlations in the fourth column are between Communication and Daily Living Skills, Communication and Socialisation, and Daily Living Skills and Socialisation, respectively. The compression of the norm-referenced metrics arises at the conversion step: for participants aged 7 to 18 years, a v-scale sum anywhere between 3 and 14 maps onto a Communication standard score of 20, and a sum of domain standard scores between 60 and 100 onto a composite of 20. The Domestic subdomain does not reach the v-scale floor because its lowest tabled value is 3 rather than 1, which is why the v-scale sums retain variance where the standard scores do not. The internal consistency of the raw domain totals is inflated by their shared dependence on age; the coefficient computed on the age-referenced v-scale sums (α = 0.756) is the one reported in the manuscript, and it applies to the measure used in the primary analysis.

Table S7. Coefficient for adaptive functioning across analytic strategies.

Analytic strategy	n	Coefficient	SE	p	FMI
Sensory seeking					
Complete cases, summed raw score	52	−0.00214	0.00353	.544	—
Multiple imputation, summed raw score	70	−0.00243	0.00356	.518	0.205
Complete cases, v-scale sum	52	−0.01380	0.01829	.451	—
Sensory avoiding					
Complete cases, summed raw score	52	−0.00421	0.00236	.074	—
Multiple imputation, summed raw score	70	−0.00411	0.00268	.140	0.244
Complete cases, v-scale sum	52	−0.00017	0.01048	.987	—

Note. The complete-case analysis on the v-scale sum is the primary analysis; the two summed-raw-score rows are reported as sensitivity analyses. Coefficients are on the logit scale and are not directly comparable in magnitude across the two measures, whose standard deviations differ (summed raw score ≈ 75 points, v-scale sum ≈ 15 points). FMI = fraction of missing information. The two measures of adaptive functioning are only moderately related (Pearson r = 0.591, Spearman ρ = 0.705). Dispersion parameters were essentially unchanged across specifications (5.19 and 5.22 for sensory seeking; 16.83 and 15.83 for sensory avoiding), indicating that the difference between strategies arises from the predictor rather than from model fit.

Table S8. Behavioural category definitions and function coding rules.

Category	Operational definition	Constituent items recorded in the dataset
**Self-injurious behaviours**	Behaviours directed at the participant's own body.	Biting oneself; hitting oneself; hitting oneself with an object; self-injury of unspecified topography; unspecified self-injurious behaviour.
**Aggression towards others**	Behaviours directed at another person's body.	Hitting; pushing a person; kicking; pinching; pulling hair; biting; scratching; grabbing; head butting; directed throwing; finger wringing; strangling; unspecified aggressive behaviour.
**Socially inappropriate behaviours**	Behaviours that breach social or institutional expectations without being directed at a person's body or at materials.	Shouting; absconding or escaping; opposing; crying; dropping to the ground; provocation; spitting; insulting; climbing; leaving an activity; removing clothes or shoes; tapping fist on table; playing with faeces; urinating in inappropriate places or on oneself; stealing objects; making repetitive requests; foot tapping; pulling; putting a hand inside one's clothing; putting feet on someone; uncooperative behaviour; throwing oneself on the floor; unspecified socially inappropriate behaviour.
**Destruction of materials**	Behaviours directed at objects or the physical environment.	Throwing materials; hitting materials; knocking down materials; breaking materials; biting an object; slamming doors; unspecified destruction of materials.
**Eating-related behaviours**	Behaviours involving the ingestion or oral exploration of edible or non-edible substances, or the seeking of food.	Pica involving oral exploration; unspecified pica behaviour; persistent food seeking; stealing food; vomiting.
**Sexualised behaviours**	Behaviours of a sexual nature enacted in a public or otherwise inappropriate context.	Touching another person inappropriately; masturbating in public; rubbing oneself against others.
**Stereotyped behaviours**	Repetitive behaviours without an apparent instrumental function. In the present records this category is populated by a single item.	Stereotyped auditory behaviour.
**Behavioural crises or meltdowns**	Episodes comprising several behaviours from different categories occurring simultaneously within a circumscribed period and attributable to a single inferred function, documented on a single record line.	Behavioural crisis or meltdown.

Note. The eight categories were developed jointly with the clinical teams by consolidating semantically equivalent labels already in use in the records, and are consistent with the classification of challenging behaviour set out by Emerson (2001). Sixty distinct items were recorded at least once across the eight categories.

Unit of analysis and a worked example of the crisis unit

Categories were applied to records as entered by practitioners at the time of observation, and episodes were not re-segmented by the research team. The unit of analysis is therefore the record line, that is, a single entry in the functional assessment file, several of which may arise from a single situation.

The crisis category is the exception. It is used routinely in the service when several behavioural topographies co-occur within a very short time window and are attributable to a single inferred function, and the whole is documented on one line. For example, a participant who, over the course of a few minutes, shouts, drops to the floor, hits an adult, and throws a chair, the practitioner attributing the sequence to a single function, generates one record coded as a behavioural crisis rather than four records coded as socially inappropriate behaviour, aggression towards others, and destruction of materials.

Two consequences follow. Mutual exclusivity holds at the level of the record but not at the level of behavioural topography, so counts for the topographical categories exclude behaviours occurring within crises and under-represent the total occurrence of those topographies. And the function attached to a crisis applies to the episode as a whole rather than to any of its constituent behaviours.

Two levels of decision in the coding of function

The functional hypothesis was recorded by the practitioner who conducted the observation, as an integral part of the functional assessment record. The assessment template used by the service specifies a set of functions from which practitioners work, so the hypotheses are not free-form interpretations, although the wording entered was not standardised. This is the first level of decision, and it belongs to the clinical setting; the research coding takes the recorded hypothesis as given and does not revisit the contingency.

The second level is the research coding itself, carried out manually and line by line by one author, without automated text matching, with the hypothesis as originally recorded retained alongside the assigned code. It comprised two operations: semantic variants designating the same function were consolidated under a single code, for example "sensory stimulation seeking" and "sensory seeking", and each record was assigned one of three codes, non-sensory, sensory seeking, or sensory avoiding.

The restrictive rule for sensory coding

A behaviour was coded as sensory-motivated only when the recorded hypothesis explicitly designated sensory seeking or sensory avoidance. In the absence of an explicit reference to the sensory domain, the hypothesis was coded as non-sensory.

The rule avoids attributing to practitioners functions they did not state, at the cost of a conservative count. Episodes whose function may well have been sensory but was recorded in non-sensory or unspecified terms were classified as non-sensory. The resulting proportion of sensory-motivated behaviours should therefore be read as a conservative estimate of the sensory functions practitioners explicitly recorded.

The additional rule applied to self-stimulation

Hypotheses designating self-stimulation do not in themselves specify a sensory function, since self-stimulation may be described for behaviours whose maintaining consequence is not sensory. An additional rule was therefore required: such hypotheses were coded as sensory seeking when the antecedent recorded for the episode referred to sensory stimulation, and as non-sensory otherwise.

The argument for the rule is that self-stimulation described in a sensory antecedent context is, in the practitioners' usage, a sensory designation in all but name, and excluding it would discard genuine cases. The argument against is that the rule imports information from the antecedent field, which the restrictive rule otherwise excludes, and thereby introduces an asymmetry: no comparable rule promotes an ambiguous hypothesis to sensory avoiding. The rule is stated here so that its effect can be assessed by readers, and the hypothesis as originally recorded was retained in the dataset so that the coding can be reproduced under an alternative rule.

Table S9. Mixed-effects logistic models of the inferred sensory function by behavioural class (random intercept for participant; reference class: socially inappropriate behaviours).

Panel A. Whether an episode was assigned a sensory function (7,560 episodes, 70 participants).

Behavioural class	OR	95% CI	p
Sexualised behaviours	4.036	2.264 to 7.196	< .0001
Eating-related behaviours	1.533	0.919 to 2.557	.102
Destruction of materials	1.042	0.778 to 1.396	.783
Aggression towards others	0.975	0.747 to 1.271	.850
Self-injurious behaviours	0.674	0.472 to 0.962	.030
Stereotyped behaviours	0.662	0.213 to 2.057	.475
Behavioural crises	0.237	0.073 to 0.770	.017

Note. Odds ratios above 1 indicate that episodes in that class were more likely than episodes in the reference class to be assigned a sensory function, and values below 1 that they were less likely; an interval containing 1 indicates that the data do not distinguish the two classes. Seven comparisons; Bonferroni-corrected threshold α = .007, which only the contrast for sexualised behaviours survives. Intraclass correlation = 0.441.

Panel B. Direction of the sensory function among the 565 sensory episodes (70 participants).

Behavioural class	OR (avoiding vs. seeking)	95% CI	p
Aggression towards others	7.420	2.623 to 20.993	.0002
Self-injurious behaviours	5.319	1.870 to 15.126	.0017
Other classes combined	3.996	0.349 to 45.749	.265
Destruction of materials	0.122	0.044 to 0.336	< .0001
Sexualised behaviours	0.029	0.003 to 0.284	.0024
Eating-related behaviours	not reported	not estimable	.0032

Note. Odds ratios above 1 indicate that sensory avoiding predominated over sensory seeking relative to the reference class, and values below 1 that sensory seeking predominated. Behavioural crises and stereotyped behaviours contributed fewer than ten sensory episodes each and were combined. Six comparisons; Bonferroni-corrected threshold α = .0083, which five of the six contrasts survive. The estimate for eating-related behaviours rests on a single episode in one cell and is not reported. Intraclass correlation = 0.762.
